# Prevalence of contagious and environmental mastitis-causing bacteria in bulk tank milk and its relationships with milking practices of dairy cattle herds in São Miguel Island (Azores)

**DOI:** 10.1007/s11250-015-0973-6

**Published:** 2015-12-30

**Authors:** Carla Azevedo, Diana Pacheco, Luísa Soares, Ricardo Romão, Mónica Moitoso, Jaime Maldonado, Roger Guix, João Simões

**Affiliations:** HIPRA, Avinguda La Selva, 135, 17170 Amer, Girona Spain; University of Évora, Largo dos colegiais 2, 7004-516 Évora, Portugal; São Miguel Young Farmers Association, Arrifes, Ponta Delgada, São Miguel, 9500-372 Azores Portugal; University of Trás-os-Montes e Alto Douro, Quinta de Prados, 5000-811 Vila Real, Portugal

**Keywords:** Cattle, Coliform bacteria, Epidemiology, Mastitis, Risk factors, *Staphylococcus aureus*

## Abstract

This study aimed to assess the degree of contamination of bulk tank milk (BTM) by *Staphylococcus* spp. and coliform bacteria and to identify major milking practices that help perpetuate them in dairy cattle herds in São Miguel Island. In July 2014, BTM was sampled and a survey concerning local milking practices was conducted on 100 herds. Semi quantitative multiplex polymerase chain reaction detected coagulase-negative staphylococci, *Escherichia coli*, *Staphylococcus aureus*, and other coliform bacteria (*Klebsiella oxytoca*, *Klebsiella pneumoniae*, and *Serratia marcescens*) in 100, 75, 59, and 35 % of BTM, respectively. According to multivariable univariate models, on herds not using hot water for cleaning the milking machine and teat liners, there was at least 3.4 more odds (*P* < 0.01) to have *S. aureus* or coliform bacteria contamination in BTM. The likelihood of finding *S. aureus* in BTM was higher (*P* < 0.001) on herds without high hygiene during milking, when milking mastitic cows at the end, on abrupt cessation of milking at dry-off, and official milk control implementation. The glove use also favored (odds ratio (OR) 5.8; *P* < 0.01) the detection of coliform bacteria in BTM. Poor milking practices identified in this study should be avoided in order to decrease *S. aureus* and coliform bacteria contamination of BTM. Other factors associated with milk quality in São Miguel Island also should be further investigated.

## Introduction

Bovine mastitis affects a high proportion of cows in dairy herds worldwide, being considered one of the major diseases causing a negative economic impact to the dairy industry (Bradley 2002). The etiopathology of cattle mastitis is multifaceted, with three main factors usually involved: exposure to microorganisms, host defense mechanisms, and environmental conditions (Zadoks et al. 2001). Information concerning prevalence and distribution of both environmental and contagious mastitis-causing bacteria (Zadoks et al. 2001; Riekerink et al. 2010), along with the identification of contributing risk factors (Leelahapongsathon et al. 2014) are crucial in order to control and/or prevent the disease.

Testing on samples from the bulk tank milk (BTM) is an accurate and effective approach for evaluating the milk quality at herd level (Cicconi-Hogan et al. 2013) and is particularly useful for the detection and identification of contagious bacteria in cows clinically affected by mastitis (Riekerink et al. 2010). Furthermore, the polymerase chain reaction (PCR) has been recently used for the specific and sensitive detection and identification of environmental and contagious mastitis-related pathogens (Katholm et al. 2012). Taponen et al. (2009) observed that the real-time PCR method (qPCR) can detect bacteria on approximately half of the negative bacteriologic cultures.

The Azores is a group of islands in the Atlantic Ocean and are an autonomous region of Portugal. The economy of Azores is mainly based on agribusiness, with the dairy industry as the most important sector employing about 17.6 % of the active population (Pinto 2010). In 2014, 51,684 dairy cows on pasture belonging to 1833 herds were officially reported in São Miguel Island (SDASM 2014). In 2013, 536,074,200 l of milk were delivered to the transformation/manufacture industry in all Azores Islands, with 65.1 % being produced in São Miguel Island (SREA 2014). Due to the high fragmentation of land in the Azores, transhumance is common for many cattle herds. Consequently, several farmers use mobile milking machines, mobile unrefrigerated tanks, and water tank trucks, resulting in a quite peculiar management system. Despite the relevance of dairy farming in San Miguel Island, to our knowledge, studies assessing the microbiological milk quality of BTM and determining milking practices and bovine mastitis in São Miguel Island have not been addressed.

The aims of this study were (a) to assess contagious and environmental bacterial contamination on BTM from dairy cow herds in São Miguel Island using qPCR method and (b) to identify major milking practices that may favor milk contamination.

## Materials and methods

### Herd selection

Herd selection covered eight regions, representing the entire territory of São Miguel Island (Table 1). At least 25 % of the herds in each region, on dependence of the local Young Farmers’ Association (http://www.ajamcja.com/) were preselected considering their size and production practices. A total of 345 dairy herds housing about 12,000 producing cows (23 % of total adults cows in the island) were considered for a preliminary screening. In order to identify independent variables for inclusion in the study, the milking procedures were recorded in a survey during veterinary visits lasting from February–June 2014.

**Table 1:** Number of farms selected according each region of S. Miguel Island (25° 30′ West longitude and 37° 50′ North latitude)

Region	Herds	
	Total number^a^	Selected
Nordeste	35	10
Povoação	30	8
Vila Franca do Campo	20	6
Lagoa	26	8
Ribeira Grande/Lomba da Maia	39	11
Ponta Delgada	79	20
Arrifes	86	27
Sete Cidades	30	10
Total	345	100

^a^Total number of farms on dependence of São Miguel Young Farmers’ Association

A representative subsample of 100 herds housing 6065 cows in production were eventually enrolled in the trial. Herd size was between 20 and 260 lactating cows, most of which (95 %) were fed exclusively on pasture. The monthly report (May 2014) delivered by the local milk processing facility was also used for the somatic cell counts (SCC) from BTM.

### Survey

A survey was developed based on previous veterinary visits, as well as on data from the National Mastitis Council (NMC) mastitis control plan (NMC 2001). It was always completed by the same interviewer during sampling and considered five topics (Table 2): (1) hygiene during milking procedures including udder, teats and tail cleanliness, the use of gloves and pre-dipping, teat and udder drying, removing foremilk, hygiene of teat cups after each milked cow, post-dipping, and milking mastitic cows only after milking all heathy females; (2) type and use of milking machine and/or tank (mobile or fixed machine and tank, cleanliness with hot or cold water); (3) mastitis diagnosis and treatment (implementation of the official milk control system, use of the California mastitis test, treatment based on antibiotic sensibility tests, veterinary assistance, and existence of treatment records); (4) dry cow period (abrupt cessation of milking, dry cow therapy of all cows, dry cow therapy according to antibiotic sensibility test, sealant use, and dry cows group); (5) calves management (calf suck its dam, colostrum administration, and calf for herd reposition). All responses were dichotomous (presence or absence) with the exception of hygiene during milking, udder and teats cleanliness, and tail cleanliness variables that were classified as low, medium, or high, according to the perception of the interviewer during the previous herd visits. Mobile BTM was also classified as present as the only collection device, present in combination with a fixed one or absent.

**Table 2:** Summary of all potential risk factors included in the different analyses

Independent variable		Recording method	Description	Breakdown category model
Milking procedures	Hygiene during milking	Visual	6-point scale^a^	High (0–2) vs. middle or low (3–5)
	Udder and teats cleanliness	Visual	4-point scale^b^	High (clean) vs. middle or low (dirty)
	Tail cleanliness	Visual	4-point scale^c^	High (1 and 2) vs. middle or low (3 and 4)
	Glove use	Interview	Whether or not gloves were used	Yes vs. no
	Pre-dipping	Interview	Whether or not the teats where dipped prior to milking	Yes vs. no
	Teats and udder drying	Interview	Whether or not the teats and udder where dried	Yes vs. no
	Elimination of first jets of milk	Interview	Whether or not the first jets of milk were eliminated	Yes vs. no
	Teat cups hygiene	Interview	Whether or not there was disinfection of teat cups between cows	Yes vs. no
	Post-dipping	Interview	Whether or not teats were dipped post milking	Yes vs. no
	Milking mastitic cows at the end	Interview	Milk mastitic cows last	Yes vs. no
Milking machine management	Mobile milking machine	Interview	Use of a mobile milking machine	Yes vs. no
	Hot water use	Interview	Use of hot water during milk routine	Yes vs. no
	Milk bulk tank	Interview	Refrigerated or mobile^d^	Refrigerated vs. mobile
Mastitis diagnosis and treatment	Official milk control implementation	DHI	Existence of official milk control	Yes vs. no
	CMT use	Interview	Use of CMT	Yes vs. no
	AST before treatment	Interview	Use of AST before treatment	Yes vs. no
	Veterinary assistance	Interview	Existence of veterinary assistance for mastitis treatment	Yes vs. no
	Treatment records	Interview	Existence of treatment records	Yes vs. no
Dry cow period	Abrupt cessation of milking at dry-off	Interview	Use of abrupt cessation of milking	Abrupt vs. gradual
	Dry cow therapy of all cows	Interview	Dry cow therapy of all cows	Yes vs. no
	Dry cow therapy according AST	Interview	Use of dry cow therapy according to AST	Yes vs. no
	Sealant use	Interview	Use of teat sealant	Yes vs. no
	Dry cows groups	Interview	Existence of dry cow group	Yes vs. no
Calves	Calf suck its dam	Interview	Calf sucks it dam	Yes vs. no
	Colostrum administration	Interview	Administration of colostrum	Yes vs. no
	For herd reposition	Interview	Calves for herd reposition	Yes vs. no

^a^According to Fregonesi and Leaver (2001): score 0—rear legs and tail, belly, clean udder; score 1—rear legs or tail with only minimal dirtiness, belly, clean udder; score 2—rear legs or tail with some dirtiness, belly, udder with minimal dirtiness; score 3—rear legs or tail dirty, belly, udder with some dirtiness; score 4—rear legs or tail very dirty, belly, udder dirty; score 5—rear legs or tail very dirty, belly, udder very dirty

^b^According to Schreiner and Ruegg (2003): clean—scores 1 (free of dirty) and 2 (slightly dirty, 2–10 % of surface area); dirty—scores 3 (moderately covered with dirt, 10–30 % of surface area) and 4 (moderately covered with caked dirt >30 % of surface area)

^c^Adapted to tail from Cook (2002): score 1—no manure present; score 2—minor splashing of manure; score 3—distinct plaques of manure; score 4—confluent plaques of manure encrusted

^d^Mobile tank: non-refrigerated milk bulk tank. In five farms, both refrigerated and non-refrigerated milk bulk tanks were used

*DHI* dairy herd improvement, *CMT* California mastitis test, *AST* antibiotic sensibility test

### Sample collection and preparation

A total of 100 BTM samples from the 100 selected herds were collected using the Startcheck® sampling kit (HIPRA, Spain) in order to test the presence of *Staphylococcus aureus*, coagulase-negative staphylococci (CNS), *Escherichia coli*, and other coliform bacteria.

Briefly, 250 μl of milk were taken from each tank, immediately after milking, using a calibrated sterile pipette. The entire sample was then transferred onto an FTA card (GE Healthcare, Barcelona, Spain), dropping it right at the center of the inoculation area (edged by a circle in the card). After drying card at room temperature (1–2 h), the sample was placed in a plastic bag containing a silica gel desiccant and then in a mailing envelope to be posted to Diagnos Laboratory (HIPRA, Spain). Upon arrival, samples were prepared for DNA extraction. Using gloves and nuclease-free material between samples, the whole inoculation area from each FTA card was cut using scissors. The circle was divided into small pieces that were transferred to a 2-ml Eppendorf tube with safety lock (to avoid cross contamination between samples) and was labelled according to the origin. After adding 1 ml of PCR nuclease-free water (Ambion® Nuclease-Free Water, Thermo Fisher Scientific, Inc., MA, USA), the tube was stirred by vortex for 15 s and then incubated 20 min at 100 °C in a dry block. Stirring was repeated, and the liquid and solid phases were separated by centrifugation for 5 s at maximum speed, according the manufacturer’s instructions. Finally, supernatant was transferred to a new tube and subjected to DNA extraction (Koskinen et al. 2009).

### DNA extraction and amplification

The extraction and amplification of the DNA were performed using the PathoProof^™^ Mastitis Complete-12 Kit (Thermo Fisher Scientific, Inc., MA, USA), with some modifications. Briefly, 600 μl of buffer AL, 60 μl of Proteinase K, and 600 μl of each supernatant were transferred to a 2-ml Eppendorf tube. The mixture was stirred with vortex for 15 s and incubated 1 h at 56 °C. Stirring was repeated for 15 s and 600 μl of ethanol was added. Subsequently, 620 ml of the mixture was transferred to an extraction column (coupled to a collection tube for each subsequent step) and centrifuged 1 min at 10,000 rpm. The latter step was repeated three times till whole mixture was passed through the column. Then 500 μl of buffer AW1 was added to the column and centrifuged 1 min at 10,000 rpm. This step was repeated with buffer AW2 but extending centrifugation to 3 min. Finally, 50 μl of buffer AE was transferred to the column, and the column was incubated 1 min at room temperature. Then the column was centrifuged 1 min at 10,000 rpm, and the eluted volume was kept frozen at−20 until further analysis.

### qPCR amplification

DNA amplification was accomplished in a Stratagene Mx3005P instrument, using the PathoProof Master and Primer mixes as per manufacturer’s instructions, in a final volume of 25 μl. A qPCR positive result was recorded when cycle threshold (Ct) values were ≤37 and a sigmoidal amplification plot was obtained. Ct values were considered as indicative of the amount of bacterial nucleic acid in a specimen, with lower values indicating higher bacterial titters. Ct values ≤30 indicated high amounts of target nucleic acid in the sample (+++), values >30 and ≤35 indicated moderate amounts (++), and values >35 and ≤37 indicated low amounts (+).

### Statistical analysis

Descriptive statistics, including the 95 % confidence intervals, were used for BTM bacteria prevalence description. Univariate univariable logistic regressions models were used considering each bacteria (nominal variable) on BTM and SCC (numerical variable). Models were considered significant at 0.05 level for likelihood ratio tests, and respective odd ratios were calculated.

In order to evaluate the influence of several practices in pathogens BTM presence, univariate multivariable logistic models were used.

Prior to building models, the multicollinearity was tested (De Vliegher et al., 2004) and an independent variable was eliminated if chi-square was >60 (1 d.f.), considering the Pearson test. Consequently, the variables “milk bulk tank” was removed and “mobile milking machine” remained in the initial model.

Each bacteria were classified as nominal variable 0 (no PCR detection) or 1 (PCR detection). All independent variables were also coded as categorical (0 or 1). Each multivariable model was building using the Hosmer and Lemeshow method (Hosmer and Lemeshow 1989). Firstly, all independent variables with *P* value <0.25 for univariate associations were included on a full model. Nonsignificant variables were successively removed and compared with the previous model, including their interactions. Finally, only the main variables (and their interactions) were considered at 0.05 level for Wald test.

The JMP® software version 7 (SAS Institute Inc., 2007) was used for all analysis.

## Results

### Prevalence of contagious and environmental pathogenics

Of the 100 samples tested, CNS were present in 100 % (95 % CI 96.3–100.0 %), *E. coli* in 75 % (95 % CI 65.782.5 %), *S. aureus* in 59 % (95 % CI 49.2–68.1 %), and coliform bacteria in 35 % (95 % CI 26.4–44.8 %). The majority of the positive samples (40–89 %) showed Ct values ≤30, indicating presence of high amounts of target nucleic acid (Table 3). *S. aureus*, *E. coli*, and coliform bacteria were concomitantly detected in 79.7 % (47/59) and 42.4 % (25/59) of BTM, respectively. Coliform bacteria and *E. coli* were simultaneously detected in 38.7 % (29/75) of BTM. All bacteria were concomitantly detected in 22 % (22/100) of the herds.

**Table 3:** Proportion of qPCR positive herds to bacteria in bulk tank milk samples

Parameter		Proportion [CI (95 %)] of herds			
		*S. aureus*	CNS^a^	*E. coli*	Coliform bacteria^b^
Negative		41.0 % (31.9–50.8 %)	–	25.0 % (17.5–34.3 %)	65.0 % (55.3–73.6 %)
Positive	>35 ≤ 37 Ct (+)	12.0 % (7.0–19.8 %)	–	21.0 % (14.2–30.0 %)	6.0 % (2.8–12.5 %)
	>30 ≤ 35 Ct (++)	42.0 % (32.8–51.8 %)	89.0 % (81.4–93.7 %)	40.0 % (30.1–49.8 %)	15.0 % (9.3–23.3 %)
	≤30 Ct (+++)	5.0 % (2.2–11.2 %)	11.0 % (6.3–18.6 %)	14.0 % (8.5–22.1 %)	14.0 % (8.5–22.1 %)

*CI (95 %)* 95 % confidence interval, *Ct* threshold cycle, (+) low amount of DNA detected, (++) moderate amount of DNA detected, (+++) high amount of DNA detected

^a^CNS (coagulase-negative staphylococci): *S. chromogenes*, *S. epidermidis*, *S. hemolyticus*, *S. saprohyticus*, *S. simulans*, *S. warneri* and *S. xylosus*

^b^Coliform bacteria: *Klebsiella oxytoca*, *Klebsiella pneumoniae* and *Serratia marcescens*

The somatic cell counts (SCC) from BTM were of ≤150,000; >150,000 ≤ 250,000; >250,000 ≤ 400,000; or ≥400,000 cells/ml in 12, 47, 36, and 5 % of all the herds, respectively. The minimum total bacterial count on BTM (≤4000 individual bacterial counts per ml) occurred in 26 % of the herds.

A significant relationship (*P* ≤ 0.001) between herds tested positive to *S. aureus* and the SCC was observed (Table 4), but not when coliform bacteria (*P* = 0.50) or *E. coli* (*P* = 0.07) BTM-positive were considered.

**Table 4:** Proportion of positive herds to *S. aureus* and their relation to somatic cell count on bulk tank milk

SCC (cells/ml)^a^	Positive herds to *S. aureus*	Odds ratio	CI (95 %)^b^	*P* value
≤150,000	16.7 % (2/12)	Reference	–	–
>150,000–≤250,000	55.3 % (26/47)	6.2	1.2–31.4	0.03
>250,000–≤400,000	72.2 % (26/36)	13.0	2.4–70.1	0.003
>400,000	100 % (5/5)	46.2	1.9–114.1	0.02

^a^SCC somatic cell count measured from bulk tank milk according official report—June 2014

^b^ *CI (95 %)* 95 % confidence interval

### Risk factors

Significant influences of several practices on the presence of bacteria on BTM were observed concerning *S. aureus* and coliform bacteria, but not *E. coli* (Table 5). Milking machine cleanliness with hot water correlated (*P* < 0.05) with the detection of both of *S. aureus* and coliform bacteria. BTM from herds without high hygiene during milking, on abrupt cessation of milking, or milking mastitic cows at the end had more chances (*P* < 0.05) to be contaminated with *S. aureus* (Table 6). The official milk control implementation (*P* < 0.05) was also related with this contagious pathogen. Glove use or “calf suck its dam” factors also influenced (*P* ≤ 0.05) the presence of coliform bacteria.

**Table 5:** Significant effect of several practices on percentage of herds affected by *S. aureus*, *E. coli*, and other coliforms in bulk tank milk using multivariable logistic regression models

Model for:	Practice (independent variable)	Positive herds without practice	Positive herds with practice (%)	*P* value^a^
*S. aureus* (Whole model: *P* < 0.001)	High hygiene during milking	74.2 % (49/66)	29.4 % (17/34)	0.007
	Hot water use	69.8 % (44/63)	40.5 % (15/37)	0.04
	Official milk control implementation	66.3 % (53/80)	30.0 % (6/20)	0.03
	Milking mastitic cows at the end	67.2 % (41/61)	46.2 % (18/39)	0.04
	Abrupt cessation of milking	67.4 % (31/46)	51.9 % (28/54)	0.04
Coliforms^b^ (Whole model: *P* < 0.001)	Glove use	40.7 % (33/81)	10.5 % (2/19)	0.05
	Hot water use	46.0 % (29/63)	16.2 % (6/37)	0.01
	Calf suck its dam	41.8 % (28/67)	21.2 % (7/33)	0.02

^a^No significant interactions between factors (independent variables) were observed (*P* > 0.05)

^b^Coliform bacteria: *Klebsiella oxytoca*, *Klebsiella pneumoniae*, and *Serratia marcescens*

**Table 6:** Odds ratios of the independent variables included in the logistic regression models

Milk pathogens	Lack of practice	Odds ratio^a^	Interval confidence 95 %	*P* value^b^
*S. aureus*	High hygiene during milking	6.9	2.8–17.4	<0.001
	Hot water use	3.4	1.5–7.9	0.004
	Official milk control implementation	4.6	1.6–13.3	0.003
	Milking mastitic cows at the end	2.4	1.1–5.5	0.04
	Abrupt cessation of milking	2.0	0.9–4.5	0.09
Coliforms^c^	Glove use	5.8	1.3–27.0	0.007
	Hot water use	4.4	1.6–12.0	0.002
	Calf suck its dam	2.7	1.0–7.0	0.04

^a^Herds with practice as referent

^b^Likelihood ratio test (1 d.f.)

^c^Coliform bacteria: *Klebsiella oxytoca*, *Klebsiella pneumoniae*, and *Serratia marcescens*

## Discussion

The microbiological milk quality is a key economic factor for dairy farms and pathogenic bacteria could arise from several sources (Berry et al. 2006; Rysánek et al. 2009; Bava et al. 2011), such as mastitic udders of cows, incrementing SCC, and/or during milking and milk transport to BTM.

The present study indicated a high prevalence of both contagious pathogen (*S. aureus*) and environmental (CNS and all coliforms) pathogens, reaching 100 % for CNS. Although CNS are considered minor pathogens with a small SCC contribution for bulk milk, unless in herds with high SCC on BTM (Schukken et al. 2009), they were considered as emerging mastitis pathogens in the last decade (Pyörälä and Taponen 2009). However, CNS prevalence at cow level can range between 0 and 100 % in herds (Schukken et al. 2009) presenting, in average, 12 % of infected cows. According to Piessens et al. (2012), environmental sources and cow-to-cow transmission appear to be involved in the epidemiology of CNS. Probably, the CNS environmental contamination of milk had an important role on the prevalence results of our study. Further researches in Azorean dairy herds are necessary in order to evaluate the role of each source and the CNS species involved (Vanderhaeghen et al. 2015).

*S. aureus*, a major pathogen related to low mastitis cure rates (Katholm et al. 2012), was detected in 59 % of the herds investigated. This finding is consistent with the prevalence ranging between 31 and 100 % in Europe and in North America (Richard et al. 2006). Katholm et al. (2012) observed the presence of *S. aureus* in 97 % of BTM from 4258 Danish herds using real-time PCR among other bacteria. A cumulative prevalence of 74 % after three successive microbial cultures on BTM samples from 258 dairy herds in Prince Edward Island (Canada) was observed by Richard et al (2006). Phuektes et al. (2003) detected *S. aureus* in 33 % of BTM from 42 Australian herds using multiplex PCR assay. In New Zealand, a country with cow management resembling that from São Miguel Island, *S. aureus* was repeatedly isolated from 57 % of the BTM by Howard (2006).

*S. aureus* can be isolated from several animal body surfaces, from the hands of the milking operator, as well as from several utensils used during milking, mainly from the teat cups, representing important transmission mechanisms (Benić et al. 2012). Moreover, *S. aureus* is frequently non-responsive to antimicrobial treatment and may remain on the udder causing a subclinical and excretory mastitis status (Katholm et al. 2012). In fact, the significant odds ratio (OR 6.2–46.2) observed in BTM with SCC higher than 150,000 cells/ml (as reference) suggests that the intramammary infections were an important source of *S. aureus* in milk in this study. Although *S. aureus* has been detected in BTM during the following month after SCC report (June 2014), this short delay and the specific epidemiologic characteristic of the mastitis provoked by this pathogen can support, at least in part, these results. Inversely, although *E. coli* was simultaneously observed in 79.7 % of BTM contaminated by *S. aureus*, no relationship was observed between global coliform occurrence and previous monthly SCC report, suggesting environmental contamination, as described by Rysanek et al. (2007).

Howard (2006) reported prevalence ratios of 51 and 11 % for CNS and coliform bacteria in BTM, respectively. Katholm et al. (2012) recorded a low prevalence ratio (13 %) of *Klebsiella* spp. (a coliform bacteria) and a high prevalence (61 %) of *E. coli*. In our study, the prevalence of environmental bacteria in BTM was higher than the reported ones in these latter studies, suggesting an overall poor milking management in several herds.

According to the multivariable analysis approach, we observed three major milking practices that influenced the presence of *S. aureus* in BTM; herds without high hygiene during milking, milking mastitic cows at the end, and hot water use for clean and disinfection of milking machine influenced the presence of this bacteria in BTM.

*S. aureus* had 2.8 to 17.4 more chance to be detected in BTM from herds with poor milking hygiene. This suggests that this poor hygiene during milking could increase the risk of intramammary infection by *S. aureus* and consequently improve the SCC in bulk milk, like the one observed in our study. Although the presence of *S. aureus* in BMT was not influenced, considering our final model, by udder/teats and tail hygiene variables, the hygiene milking indicators (rear legs, tail, belly, and udder) used by in our study was extensive to other surfaces of body parts that can contaminate milk during milking. In fact, Schreiner and Ruegg (2004) described that *S. aureus* in milk was more frequently isolated from cows under poor udder hygiene. According to these authors, the association between udder hygiene status and contagious mastitis may indicate that control methods for contagious mastitis (e.g., teat dipping and sanitation) are not as effective as expected when udders are dirty. Piccinini et al. (2009) isolated specific strains of *S. aureus* not only from milk but also from the teat skin, suggesting their active role in the epidemiology of the mastitis. Concerning tail cleanliness, several authors were unable to establish a significant improvement in cow udder cleanliness or udder health that could be attributed to tail docking, seeing no advantage in adopting this procedure in order to improve milk quality (Eicher et al. 2001; Tucker et al. 2001; Schreiner and Ruegg 2002; Ruegg 2004).

Milking mastitic cows in no specific order was another practice that significantly affected the *S. aureus* presence in BTM in this study. Riekerink et al. (2006) also observed lower isolation rates for *S. aureus* in BTM belonging to herds with mastitis-affected cows milked separately from healthy females. Teat cups were considered the main transmission vehicle (Benić et al. 2012), and therefore, affected cows should be milked separately or after healthy animals (NMC 2001; Zecconi 2006; Arnold and Bewley 2011; Benić et al. 2012; Middleton 2013).

Milking machine cleanliness and hygiene is a critical point that influences bacterial counts in BTM (Bava et al. 2009; Biggs 2009). In our study, herds without hot water use was 3.4 and 4.4 more likely (*P* ≤ 0.01) to present *S. aureus* and coliform bacteria in BTM, respectively. Bava et al. (2009) observed an increase of coliform bacteria counts in BTM when washing with detergent was performed at <40 °C. Adequate water temperature is an important factor for milk fat (solidifying at <35 °C) residue removal from the surfaces of the milking equipment (Monken and Ingalls 2002). Also, the cleaning solution activity increases with warm water (Reinemann et al. 2003).

An interesting result in our study was the positive influence of abrupt cessation of milking at dry-off on the presence of *S. aureus* in BTM. The abrupt cessation of milking is a generalized practice in association with antimicrobial intramammary administrations and teat sealants. However, it was recently reported (Zobel et al. 2013) that the gradual cessation of milk in high producing dairy cows could be benefic for animal welfare such as udder engorgement, milk leakage, and lying behavior. Nevertheless, Rajala-Schultz et al. (2005) found that increasing milk yield at dry-off has an effect on the incidence of environmental mastitis but not on other pathogens such as CNS.

Herds without official milk control implementation had 4.6 more chance to present *S. aureus* in bulk milk, and this suggests that this tool is very important for mastitis management. According to the NMC mastitis control plan (NMC 2001), periodic reports regarding affected quarters, individual CCS, time of disease occurrence, antibiotic sensitivity data sets, and the use reports of subclinical and/or clinical mastitis are crucial for overall mastitis control assessment (Wenz 2004).

In the present study, other than the hot water use, the use of gloves and the “calf suck its dam” influenced the presence of coliform bacteria on BTM. The use of gloves is one of the most important measures to prevent mastitis caused by coliform (and *S. aureus*) agents in dairy cows (Petersson-Wolfe et al. 2010; Arnold and Bewley 2011) and are included in the NMC mastitis control plan (NMC 2001). The operator’s hands can be a vehicle for bacteria dissemination, and gloves can decrease this spread due to the lower bacteria adhesion to plastic or caoutchouc surfaces (Nickerson 2014). Dufour et al. (2012) observed a decrease of *S. aureus* mastitis prevalence and incidence, even between quarters, when gloves were used by operators. In our study, similar findings were observed for coliform bacteria occurrence in BTM. Concerning the impact of calf suckling, our results are in agreement with the findings of González-Sedano et al. (2010) after evaluation of residual calf suckling effect on clinical and subclinical infections of mastitis in dual-purpose cows concluding that eliminating this practice presents a high risk for development of mastitis. In fact, in high-producing dairy cows, suckling decreases the risk of mastitis in the suckling period and in some cows even for some time after the suckling has been terminated (Krohn 2001).

In conclusion, SCN, S*. aureus*, and coliform bacteria contamination of BTM from dairy cow herds of São Miguel Island was prevalent. Hot water machine cleanliness seems to be a major factor that increases the chance for both bacteria to be present in BTM. Herds without practices such as high hygiene during milking udder and milking mastitic cows at the end also had more chances to present *S. aureus* in BTM. Coliform bacteria in BTM were also more prevalent in herds without glove use and when calf does not suck its dam. These findings, together with the monitoring of dairy herds conducted by the local plan for control of bovine mastitis, can help to improve the health status of the dairy industry on the island São Miguel Island.
